# Delayed intrabiliary migration of embolization material after hepatic arterial embolization: a case report

**DOI:** 10.3389/fsurg.2026.1842770

**Published:** 2026-07-16

**Authors:** Xiudi Chen, Yukun Ying, Hongwei Qian, Zhihong Shen

**Affiliations:** 1 Department of Hepatobiliary and Pancreatic Surgery, Shaoxing People's Hospital, Shaoxing, China; 2 School of Medicine, Shaoxing University, Shaoxing, Zhejiang, China; 3 Shaoxing Key Laboratory of Minimally Invasive Abdominal Surgery and Precise Treatment of Tumor, Shaoxing, China

**Keywords:** case report, choledocholithiasis, ERCP, hepatic artery embolization, migration

## Abstract

**Background:**

Transcatheter arterial embolization (TAE) using metallic coils is a widely accepted treatment for arterial bleeding in hepatobiliary surgery. Although generally safe, delayed migration of embolization materials into the biliary system is a rare but clinically significant complication that may lead to recurrent biliary symptoms. Early diagnosis can be challenging because of anatomical overlap and imaging limitations.

**Case presentation:**

We report a 73-year-old man with recurrent choledocholithiasis and cholangitis following right hepatic artery embolization with metallic coils. After laparoscopic cholecystectomy and common bile duct exploration complicated by postoperative hemorrhage, TAE was performed successfully. Two years later, the patient developed recurrent biliary symptoms and underwent multiple endoscopic retrograde cholangiopancreatography (ERCP) procedures. Post-procedural T-tube cholangiography and subsequent ERCP demonstrated bile duct dilatation and persistent metallic shadows in the hepatic hilum; however, definitive intrabiliary localization of the embolization material could not be established because of anatomical overlap and the absence of a clear filling defect. During a later ERCP for recurrent cholangitis, a metallic wire–like structure was partially extracted under direct endoscopic visualization, confirming intrabiliary involvement of embolization material. Further removal was withheld because of resistance during traction and concern for vascular injury.

**Conclusion:**

This case illustrates the gradual and diagnostically ambiguous nature of intrabiliary migration of embolization materials following hepatic arterial embolization. Repeated evaluation over time and cautious endoscopic decision-making may be necessary when recurrent biliary symptoms occur after embolization.

## Introduction

Transcatheter arterial embolization (TAE) using metallic coils is a well-established and effective intervention for the management of arterial bleeding and pseudoaneurysms in hepatobiliary surgery (1). Although generally considered safe, delayed complications related to embolization materials have been increasingly recognized, among which migration of coils into adjacent organs is rare but clinically significant (2).

Intrabiliary migration of embolization coils has been sporadically reported and may result in biliary obstruction, recurrent cholangitis, pancreatitis, or secondary choledocholithiasis (3, 4). In most published case reports, the diagnosis is described as being established at a single time point, often based on cross-sectional imaging or endoscopic findings, followed by immediate endoscopic or surgical removal of the migrated material. However, such reports tend to emphasize definitive localization, while the diagnostic uncertainty and dynamic nature of coil migration are rarely addressed.

In clinical practice, precise localization of migrated embolization materials within the biliary system can be challenging. Metallic artifacts on computed tomography, combined with the close anatomical relationship between the hepatic arterial branches and the common bile duct at the hepatic hilum, may significantly limit the diagnostic accuracy of imaging modalities (5). Even endoscopic retrograde cholangiopancreatography (ERCP), although regarded as the most informative tool for biliary evaluation, may fail to identify small metallic components that do not produce a clear filling defect, particularly in the setting of biliary dilatation or inflammation (6).

Here, we report a case of recurrent choledocholithiasis and cholangitis following right hepatic artery embolization, in which suspected intrabiliary coil migration could not be definitively confirmed by imaging or ERCP at an early stage. Through longitudinal documentation of multiple ERCP procedures and eventual partial extraction of a metallic wire during stone retrieval, this case highlights diagnostic blind spots, temporal evolution, and real-world clinical dilemmas associated with intrabiliary coil migration.

## Case presentation

A 73-year-old male patient was admitted for symptomatic cholelithiasis accompanied by choledocholithiasis. Laparoscopic cholecystectomy combined with laparoscopic common bile duct exploration (LC + LCBDE) was performed. During the operation, a cholecysto-colonic fistula was identified and divided using a linear stapling device.

**Figure 1:**
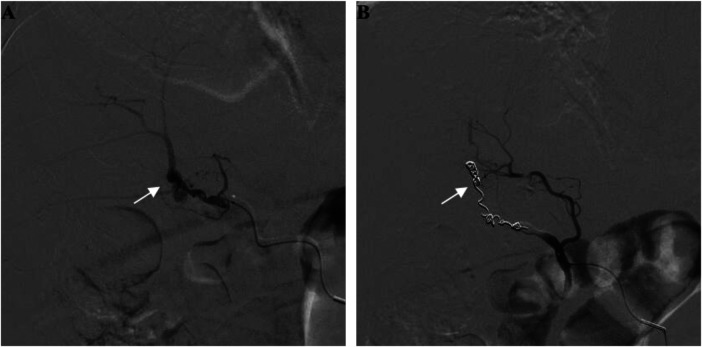
Angiographic findings during transcatheter arterial embolization. **(A)** Selective angiography demonstrating a pseudoaneurysm arising from the right hepatic artery (arrow). **(B)** Completion angiography after embolization with multiple metallic coils (arrow), showing successful occlusion of the pseudoaneurysm.

Postoperatively, the patient developed intra-abdominal hemorrhage. Computed tomography suggested a pseudoaneurysm of the right hepatic artery. Subsequent digital subtraction angiography (DSA) demonstrated a right hepatic artery pseudoaneurysm with focal contrast extravasation and contrast retention. Emergency transcatheter arterial embolization was performed via a superselective approach using seven metallic coils (three 4-mm coils, two 5-mm coils, and two 3-mm coils) supplemented with gelatin sponge particles. Completion angiography confirmed successful occlusion of the pseudoaneurysm without further contrast extravasation (Figure 1). The patient recovered uneventfully and was discharged.

**Figure 2:**
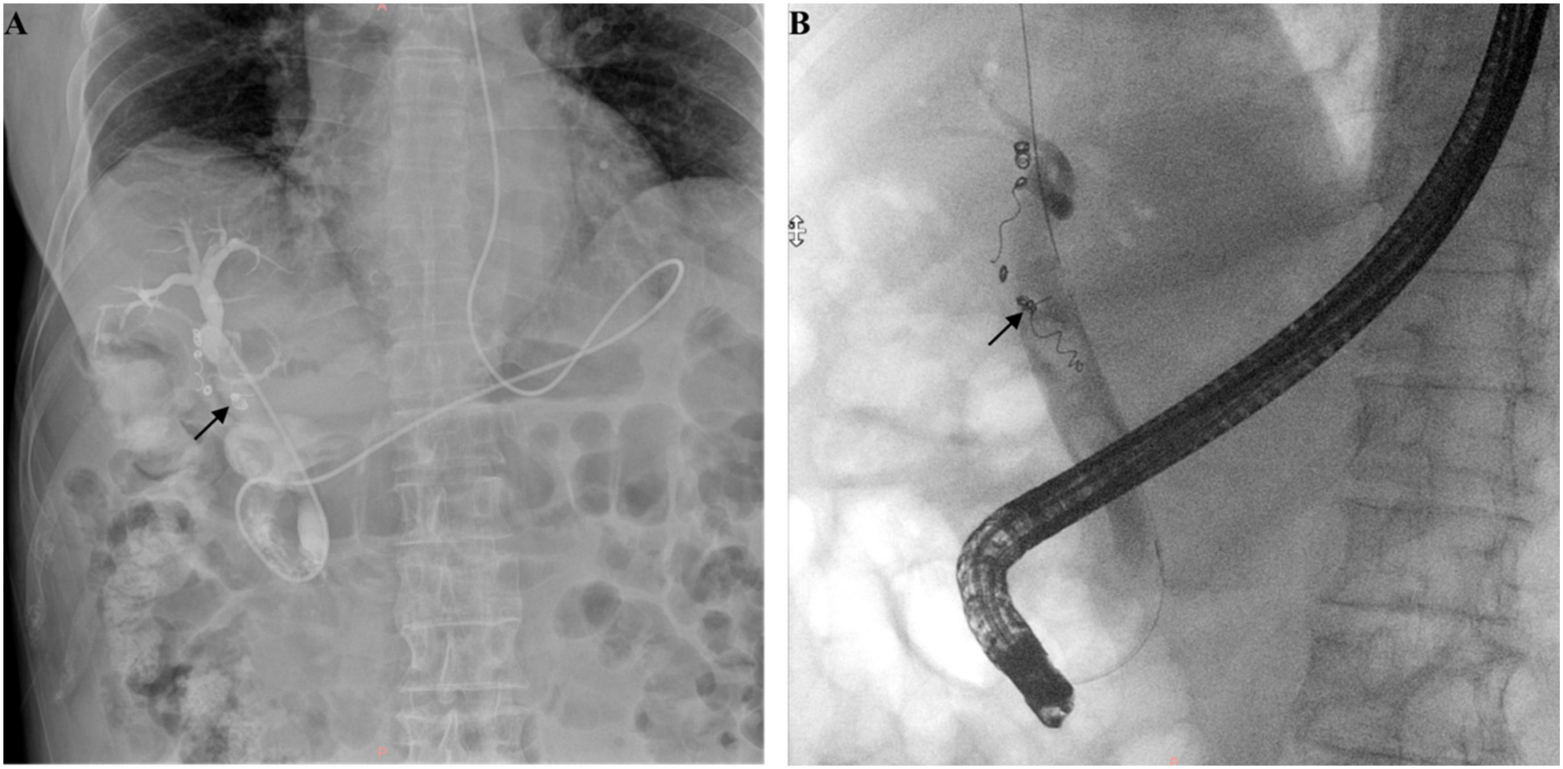
Longitudinal cholangiographic and endoscopic documentation of embolization coil–related biliary findings. **(A)** T-tube cholangiography performed after the first episode of recurrent choledocholithiasis and endoscopic treatment. The cholangiogram demonstrates a dilated biliary tree without a definite intraductal filling defect. Embolization material from prior right hepatic artery embolization is visible in the hepatic hilum (arrow); however, its precise relationship to the bile duct lumen cannot be definitively determined because of anatomical overlap between the hepatic artery and the biliary system. **(B)** Endoscopic retrograde cholangiopancreatography (ERCP) during the subsequent episode of recurrent cholangitis. Fluoroscopic imaging obtained during stone extraction demonstrates intraluminal exposure of a metallic wire–like structure (arrow), which was partially retrieved under direct endoscopic visualization and confirmed to be embolization material.

Two years later, the patient was readmitted with recurrent choledocholithiasis complicated by acute cholangitis. ERCP was performed for biliary drainage and stone removal. Post-procedural T-tube cholangiography demonstrated diffuse biliary dilatation without a definite intraductal filling defect. A metallic density corresponding to prior embolization material was visible in the hepatic hilum; however, its exact relationship to the bile duct lumen could not be determined because of anatomical overlap between the biliary tree and adjacent vascular structures (Figure 2A).

Approximately six months later, the patient again presented with recurrent choledocholithiasis and cholangitis and underwent repeat ERCP. Fluoroscopic imaging revealed persistent metallic shadows projected over the hepatic hilum (Figure 2B). Despite recurrent biliary symptoms, no definite filling defect or direct evidence of intrabiliary migration could be identified, and precise localization of the embolization material remained inconclusive because of anatomical overlap between the right hepatic artery and the common bile duct.

**Figure 3:**
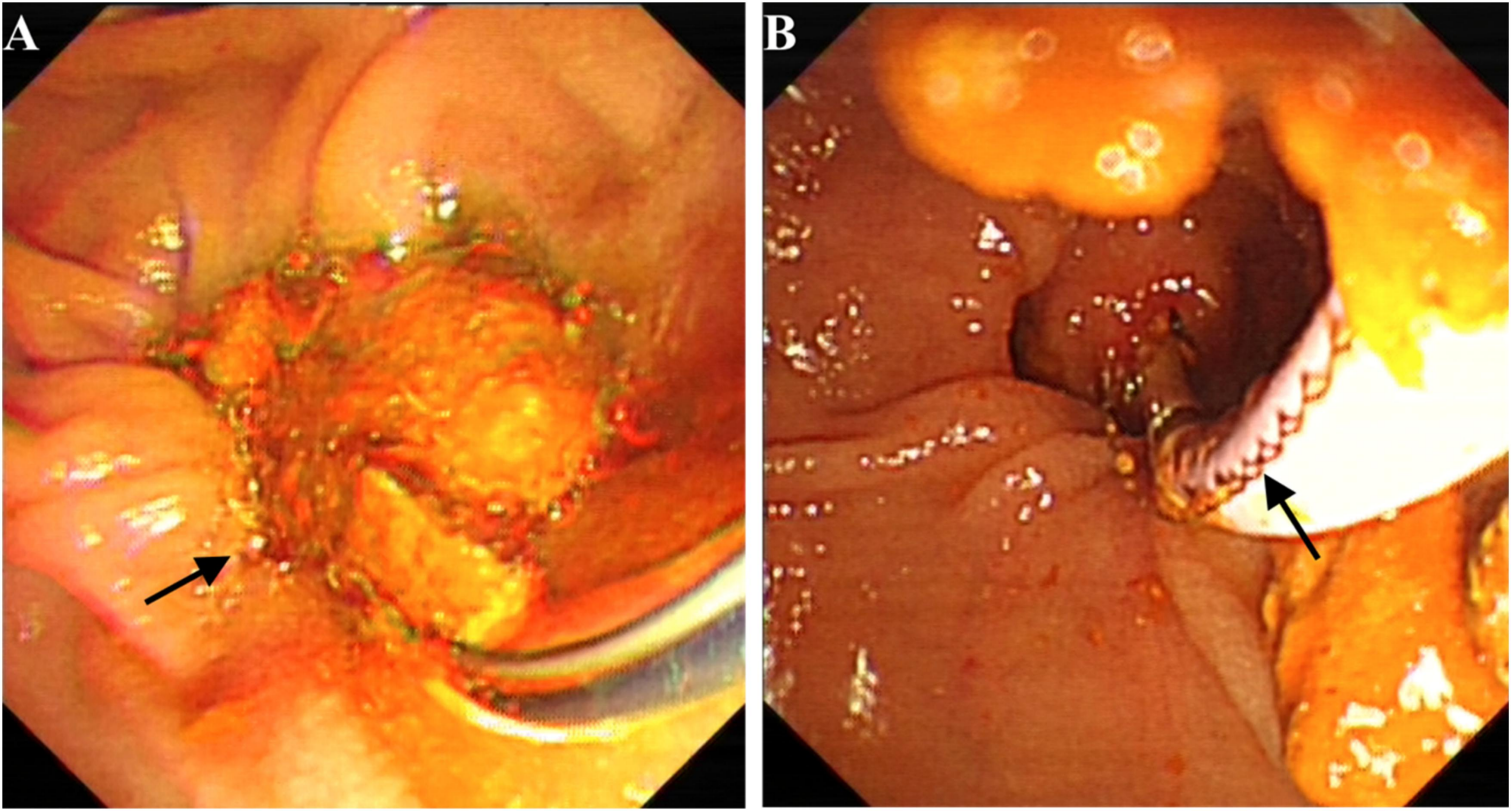
Endoscopic visualization of intrabiliary embolization material during ERCP. **(A)** Endoscopic view during stone extraction demonstrates exposure of embolization material within the bile duct lumen (arrow), partially embedded in biliary debris and inflammatory material. **(B)** Following retrieval of the embolization material, an additional metallic wire–like structure becomes visible within the bile duct (arrow), indicating the presence of residual embolization material after initial extraction.

During endoscopic stone extraction, a metallic wire–like structure was partially pulled out through the bile duct. Direct endoscopic visualization confirmed that the metallic wire–like structure was protruding into the bile duct lumen (Figure 3A), providing the first definitive evidence of intrabiliary involvement of the embolization material. An embolization material was initially retrieved, followed by partial extraction of an additional metallic wire–like structure. However, further traction encountered significant resistance, and complete mobilization of the material could not be achieved. Given the inability to confirm complete intrabiliary detachment and the potential risk of vascular injury, the procedure was intentionally terminated. Following partial extraction, residual embolization material remained visible within the bile duct (Figure 3B), raising concern regarding incomplete intrabiliary detachment and potential vascular continuity.

Because the remaining material appeared firmly embedded and further extraction was considered to carry a potential risk of vascular injury, no additional endoscopic or surgical intervention was performed. The patient was subsequently managed conservatively with regular outpatient follow-up. During over one year of follow-up, the patient remained asymptomatic without recurrent cholangitis, biliary obstruction, or choledocholithiasis.

Throughout the diagnostic process, neither cross-sectional imaging nor ERCP was able to definitively confirm intrabiliary migration of the embolization material at an early stage. It also remained uncertain whether migration into the bile duct had already occurred at the time of the first ERCP. Definitive evidence of biliary involvement was obtained only during the final ERCP, when embolization material was partially extracted under direct endoscopic visualization. The clinical course and key management steps are summarized in Table 1.

**Table 1:** Timeline of the clinical course.

Time	Clinical event	Key findings/management
T0	Initial admission and surgery	Symptomatic cholelithiasis with choledocholithiasis; LC + LCBDE performed; cholecysto-colonic fistula divided.
Early postoperative period	Postoperative hemorrhage and embolization	Intra-abdominal hemorrhage secondary to a right hepatic artery pseudoaneurysm. DSA demonstrated contrast extravasation and pseudoaneurysm formation. Superselective transcatheter arterial embolization (TAE) was performed using seven metallic coils and gelatin sponge particles, achieving complete occlusion of the pseudoaneurysm.
Approximately 2 years after embolization	First recurrent biliary episode	Recurrent choledocholithiasis with acute cholangitis; ERCP stone extraction. T-tube cholangiography showed biliary dilatation and a metallic shadow at the hepatic hilum without a definite filling defect.
Approximately 2 years 6 months after embolization	Subsequent recurrent episode and repeat ERCP	Persistent metallic shadows remained difficult to localize because of anatomical overlap.
Same ERCP	Definitive intrabiliary confirmation	A metallic wire-like structure was exposed during stone extraction and partially retrieved under direct endoscopic visualization. Further traction was stopped because of resistance and concern for vascular injury.
After partial extraction	Final management and follow-up	No additional endoscopic or surgical intervention was performed. The patient underwent conservative management with regular follow-up and remained free of recurrent cholangitis, biliary obstruction, or choledocholithiasis during more than one year of follow-up.

## Discussion

Migration of embolization material into the biliary system is an uncommon but increasingly recognized delayed complication following transcatheter arterial embolization. Several mechanisms have been proposed, including gradual erosion of the vessel wall, formation of arterio-biliary fistulas, local ischemia, and chronic inflammation (7, 8). In the present case, prior biliary surgery, postoperative hemorrhage, and subsequent right hepatic artery embolization may have collectively contributed to local tissue injury, predisposing the embolization material to delayed migration toward adjacent biliary structures.

Clinically, intrabiliary migration of embolization materials has been associated with recurrent cholangitis, pancreatitis, and secondary choledocholithiasis, often with the migrated material acting as a nidus for stone formation (4). Previously reported cases of embolization coil or material migration into the biliary system are summarized in Table 2. Most of these cases described intrabiliary migration as a discrete event diagnosed at a single time point, supported by clear imaging or endoscopic evidence, sometimes with calculi encasing the migrated material. In contrast, the present case demonstrates a prolonged period of diagnostic uncertainty despite repeated imaging and ERCP, suggesting that intrabiliary migration of embolization material may represent a gradual and dynamic process rather than an abrupt event. Notably, recurrent choledocholithiasis occurred before definitive intrabiliary involvement of the embolization material was confirmed, and no imaging or endoscopic evidence demonstrated stone formation surrounding the coil. These observations indicate that the temporal and causal relationship between embolization material migration and biliary stone formation may be more complex and variable than previously assumed.

**Table 2:** Reported cases of embolization coil/material migration into the biliary system.

Author	Year	Embolization Site	Migration Site	Clinical Presentation	Diagnostic Modality	Treatment	Key Features
Chen et al. (Present)	2026	Right hepatic artery	Common bile duct	Recurrent choledocholithiasis, cholangitis	T-tube cholangiography, ERCP, endoscopy	Partial endoscopic retrieval	Multiple ERCPs; prolonged diagnostic uncertainty; diagnosis confirmed during endoscopic retrieval; extraction intentionally halted because of vascular injury concern
Hirata et al. (16)	2026	Right hepatic artery aneurysm	Bile duct	Cholangitis, biliary obstruction, liver abscesses	Peroral cholangioscopy	Endoscopic removal via cholangioscopy	First cholangioscopy-guided removal report
Edmunds et al. (8)	2025	Hepatic artery pseudoaneurysm	Bile duct	Abdominal pain, jaundice	CT, ERCP	Endoscopic removal	Coil migration secondary to biliary stent erosion
Funamizu et al. (4)	2024	Vascular embolization	Intrahepatic bile duct	Recurrent cholangitis	CT, ERCP	Endoscopic lithotripsy + coil removal	Intrahepatic calculus formed around migrated coil
Slim et al. (17)	2023	Hepatic artery pseudoaneurysm	Common bile duct	Recurrent cholangitis with biliary stones	ERCP	Endoscopic coil extraction	Repeated ERCPs over 2 years; coil emerged through papilla during stone extraction
Elsayed et al. (15)	2023	Hepatic artery pseudoaneurysm	Biliary tree	Post-prandial pain, recurrent cholangitis	Fluoroscopy + percutaneous cholangioscopy	Percutaneous transhepatic retrieval	Non-standard access; coils eroded over years
Kao et al. (7)	2011	Hepatic artery pseudoaneurysm	Common bile duct	Obstructive jaundice	ERCP	Endoscopic removal	Delayed migration (8 years); coil embedded within CBD stone
Zuberi et al. (13)	2018	Gastroduodenal artery pseudoaneurysm	CBD + duodenum	Biliary obstruction	CT, endoscopy	Choledochojejunostomy	CBD-duodenal fistula; required surgical bypass
Zaafouri et al. (3)	2017	Gastroduodenal artery pseudoaneurysm	Common bile duct	Ascending cholangitis	CT, ERCP	Endoscopic removal	Coil as nidus for stone formation
Taibi and Legros	2019	Right hepatic artery	Common bile duct	Cholangitis, biliary leakage after cholecystectomy	CT, MRI, ERCP	Endoscopic management during biliary stent removal	Vasculobiliary injury; coil migration detected 3 months after embolization
Ozkan et al. (9)	2002	Hepatic artery pseudoaneurysm	Common bile duct	Pancreatitis	CT, ERCP	Surgical CBD exploration and coil removal	Delayed migration (2 years); coil erosion into CBD causing pancreatitis; required surgery

A key feature of this case is the diagnostic challenge encountered despite repeated imaging and endoscopic evaluation. Cross-sectional imaging was limited by metallic artifacts and the close anatomical relationship between the right hepatic artery and the common bile duct at the hepatic hilum. Even ERCP, which is generally regarded as the most informative modality for biliary pathology, failed to definitively confirm intrabiliary migration at an early stage. The small caliber of the metallic wire and the absence of a clear filling defect further reduced the diagnostic sensitivity of fluoroscopic cholangiography, particularly in the setting of bile duct dilatation and inflammation.

Importantly, definitive evidence of biliary involvement was obtained only during the final ERCP, when a metallic wire–like structure was partially extracted under direct endoscopic visualization. This observation highlights a diagnostic blind spot in current clinical practice: intrabiliary migration of embolization materials may remain occult until mechanical interaction occurs during endoscopic intervention. Our longitudinal documentation through multiple ERCP procedures underscores the limitations of relying on a single diagnostic modality or time point to establish the diagnosis.

Management of migrated embolization materials within the biliary system is not standardized. Endoscopic removal is generally preferred when feasible, while surgical or radiological intervention may be required in selected cases. In the present case, although partial endoscopic retrieval was achieved, further extraction was intentionally withheld because of resistance during traction and the inability to confirm complete intrabiliary detachment. This decision reflects a cautious, safety-oriented approach in the setting of uncertain anatomical relationships rather than technical failure.

From a practical perspective, intrabiliary migration of embolization material should be considered in patients with a history of hepatic or gastroduodenal arterial embolization who subsequently develop recurrent cholangitis, otherwise unexplained biliary obstruction or pancreatitis, recurrent choledocholithiasis, or hemobilia (9–14). Cross-sectional imaging may be nondiagnostic because beam-hardening artifacts and the close spatial relationship between hilar vessels and bile ducts can obscure the exact compartment of the material; a static study also cannot reliably distinguish an extrabiliary coil abutting the duct from a partially eroded component (9, 13, 15). ERCP provides a luminal outline rather than direct visualization of the entire duct wall and may therefore miss a thin or partially embedded metallic strand when no clear filling defect is produced. Accordingly, a negative single examination should not exclude migration when the clinical pattern persists. Comparison of serial imaging, multidisciplinary review of the original embolization anatomy, and repeat biliary assessment—potentially including cholangioscopy when feasible—may be more informative than reliance on one modality or time point (15, 16). If endoscopic retrieval is attempted, the anticipated benefit of removing an infectious or lithogenic foreign body should be balanced against the possibility that the material remains attached to, or traverses, a patent vessel (16). Review of prior angiography and current vascular imaging, availability of interventional radiology or surgical support, and cessation of traction when substantial resistance is encountered are prudent safeguards. When complete intrabiliary detachment cannot be established, staged evaluation or partial retrieval may represent a reasonable alternative to forceful extraction in selected patients.

In summary, this case highlights the challenges of diagnosing intrabiliary migration of embolization material when imaging and ERCP findings remain inconclusive. Persistent biliary symptoms after arterial embolization should prompt continued reassessment, and careful endoscopic decision-making is essential when complete intrabiliary detachment cannot be established.

## Data Availability

The original contributions presented in the study are included in the article/Supplementary Material, further inquiries can be directed to the corresponding author.
